# Statistical Optimization of Ultraviolet Irradiate Conditions for Vitamin D_2_ Synthesis in Oyster Mushrooms (*Pleurotus ostreatus*) Using Response Surface Methodology

**DOI:** 10.1371/journal.pone.0095359

**Published:** 2014-04-15

**Authors:** Wei-Jie Wu, Byung-Yong Ahn

**Affiliations:** 1 Department of Food Science & Biotechnology, Chonbuk National University, Iksan, Republic of Korea; 2 Department of Oriental Medicine Resources, Chonbuk National University, Iksan, Republic of Korea; University of Tennessee, United States of America

## Abstract

Response surface methodology (RSM) was used to determine the optimum vitamin D_2_ synthesis conditions in oyster mushrooms (*Pleurotus ostreatus*). Ultraviolet B (UV-B) was selected as the most efficient irradiation source for the preliminary experiment, in addition to the levels of three independent variables, which included ambient temperature (25–45°C), exposure time (40–120 min), and irradiation intensity (0.6–1.2 W/m^2^). The statistical analysis indicated that, for the range which was studied, irradiation intensity was the most critical factor that affected vitamin D_2_ synthesis in oyster mushrooms. Under optimal conditions (ambient temperature of 28.16°C, UV-B intensity of 1.14 W/m^2^, and exposure time of 94.28 min), the experimental vitamin D_2_ content of 239.67 µg/g (dry weight) was in very good agreement with the predicted value of 245.49 µg/g, which verified the practicability of this strategy. Compared to fresh mushrooms, the lyophilized mushroom powder can synthesize remarkably higher level of vitamin D_2_ (498.10 µg/g) within much shorter UV-B exposure time (10 min), and thus should receive attention from the food processing industry.

## Introduction

Vitamin D is essential for human health, and its deficiency is currently an unrecognized epidemic which is associated with common cancers, rickets, osteoporosis, diabetes, autoimmune diseases, and cardiovascular disease [1], [2]. Exposure to sunlight is the primary source of vitamin D for humans, however this has become problematic due to wearing sunscreen, obesity, melanin, and aging, which interfere with solar exposure and can lead to vitamin D deficiency [3], [4]. Hence, there is an urgent need for an alternative dietary source of vitamin D to protect people from such a deficiency. Unfortunately, natural food sources for vitamin D are limited to seafood, mushrooms, and egg yolks [4].

In nature, there are two distinct forms of vitamin D: vitamin D_2_ and vitamin D_3_. Vitamin D_2_, known as “ergocalciferol”, is created when ergosterol is activated by UV irradiation; where ergosterol as the precursor of vitamin D_2_ can be obtained environmentally from yeast and mushrooms [5]. Vitamin D_3_ (cholecalciferol) mainly exists in animal products such as fish liver oils, fish, butter, margarine and cheese [6]. Some studies have suggested that vitamin D_3_ is more effective than vitamin D_2_ in humans [7], [8], however, recent researches have reported that vitamin D_2_ is as effective as vitamin D_3_ in maintaining circulating concentrations of 25-hydroxyvitamin D and improving bone health [5], [9]. Currently, vitamin D_3_ supplements are derived from animal sources, which are problematic for vegetarians, for whom it is recommended to utilize the vitamin D_2_ form [4]. Moreover, the therapeutic application of vitamin D_3_ analogues may be limited due to their hypercalcemic effects [10]. Therefore, studies that broaden the clinical supplications of vitamin D_2_ merit further attention.

Wild mushrooms are considered as the only non-animal-based natural food source containing vitamin D_2_ which is converted from ergosterol [11]. Cultivated mushroom are deficient in vitamin D_2_; however, they are a rich source of ergosterol, and the vitamin D_2_ concentration could be improved by exposure to ultraviolet light [3], [10], [12], [13]. Although several studies have investigated vitamin D_2_ synthesis via UV irradiation, all of the previous studies determined the optimal operating conditions (irradiation temperature, exposure time, and irradiation intensity) by varying one parameter and keeping the other parameters constant [3], [6], [12]. The fatal disadvantage of this single-variable optimization is that it does not include interactive effects that occur among the variables, and it cannot depict the complete effects of various parameters on the conversion rate [14]. Therefore, attempts to replicate these results in commercial environments have been impractical for mushroom producers due to the variable amounts of vitamin D_2_ that are produced from fickle UV irradiation intensities, and exposure time [3]. These challenges could be overcome with optimization studies that use respond surface methodology (RSM).

Respond surface methodology (RSM) is a collection of statistical and mathematical techniques that optimize responses. This technique is useful for developing, improving and optimizing processes in which a response of interest is influenced by several variables [14]. RSM minimizes the number of experimental trials needed to evaluate the variables interactive effects, and has been successfully employed to model and optimize biotechnological and biochemical processes [15], [16].

The objective of the present study was to establish the optimal UV irradiation operation conditions needed to maximize the vitamin D_2_ yield in oyster mushrooms using RSM, and ultimately improve the practical application in the food industry.

## Materials and Methods

### Ethics statement

Fresh oyster mushrooms (*Pleurotus ostreatus*) were purchased from a local grower, and this mushroom also does not come under “endangered or protected species”, hence this study does not require any permission.

### Chemicals and materials

Ergosterol and vitamin D_2_ standards were purchased from Sigma-Aldrich (St. Louis, MO, USA). Potassium hydroxide (AR grade), sodium *L*-ascorbate (AR grade), *n*-pentane (extra pure), and 95% ethanol (extra pure) were obtained from Samchun Pure Chemical (Pyeongtaek, Korea). The methanol, ethanol, and acetonitrile solvents were of HPLC grade and purchased from J.T. Baker (Avantor Performance Materials, Center Valley, PA, USA). Fresh oyster mushrooms (*Pleurotus ostreatus*) were obtained from a local grower, and were used immediately. Irradiation was performed with the following UV lamps: UV-A lamps (Model F15T8BL, Sankyo Denki, Japan) with highly concentrated radiation between 315 and 400 nm (at a peak of 352 nm), UV-B lamps (Model G15T8E, Sankyo Denki, Japan) that emitted ultraviolet rays between 280 and 360 nm (at a peak of 306 nm), and UV-C lamps (Model G15T8-AN, Sankyo Denki, Japan) with a maximum light intensity output of 253.7 nm.

### UV irradiation wavelength effects on vitamin D_2_ synthesis in oyster mushrooms

This study investigated the vitamin D_2_ synthesis efficiency of oyster mushrooms, when exposed to different wavelength bands of ultraviolet irradiation. Fresh oyster mushrooms were subjected to different sources of irradiation with UV-A, UV-B, UV-C, UV-A & B, UV-A & C, UV-B & C, or UV-A & B & C lights, respectively. The irradiation intensity was measured with a StellarNet BLACK-Comet-CXR spectrometer (CXR-SR-50, StellarNet Inc., Tampa, FL, USA USA) set to 0.6 W/m^2^, at an ambient temperature of 35°C. The mushroom irradiation procedures were performed with their gills facing the UV source for 60 min, and then the specimens were irradiated for another 60 min with the caps facing the UV source. After irradiation, samples were immediately frozen at −70°C and lyophilized separately, then homogenized with a pulverizator (RT-08, Rong Tsong, Taiwan) to prepare for the analysis. Based on the synthesized vitamin D_2_ content in the mushrooms, the optimal light source was selected for further study.

### Selection of irradiation conditions

The initial step of the preliminary experiment was to select an appropriate temperature for vitamin D_2_ synthesis. The ambient temperatures were ranged from 15 to 45°C, with the irradiation intensity and exposure time at 0.6 W/m^2^ and 60 min for each side, respectively. While the second step of the preliminary experiment was to determine the exposure time. Using the optimal ambient temperature from the previous step, each side of the mushrooms was exposed to UV irradiation for different time periods, which varied from 20 to 100 min, with a consistent irradiation intensity of 0.6 W/m^2^. Final step of the preliminary experiment was to select the appropriate UV irradiation intensity for vitamin D_2_ conversion. Radiation exposures were performed at the optimal ambient temperature determined in the first step, the appropriate exposure time from the second step, and a UV irradiation intensity that were differed from 0.3 to 1.2 W/m^2^. Based on the results, the three levels (lower, middle, upper) of each process variable were determined for RSM.

### Vitamin D_2_ and ergosterol analysis

Vitamin D_2_ and ergosterol were extracted and analyzed according to the method of Koyyalamudi et al. [3], with slight modifications. Freeze-dried mushroom sample powder (1 g) was mixed with 50 mL of ethanol (95%), 4 mL of sodium ascorbate solution (17.5 g of sodium ascorbate in 100 mL of 1 M NaOH), and 10 mL of 50% potassium hydroxide. The mixture was saponified under reflux at 80°C for 1 h, then extracted with 15 mL of de-ionized water and 15 mL of ethanol, followed by three stages of *n*-pentane with volumes of 50, 50, and 20 mL, respectively. The *n*-pentane layers were pooled and washed three times with 3% KOH in 5% ethanol and then with de-ionized water until neutralized. The organic layer was rotary evaporated (EYELA, Rikakikai Co., Ltd. Tokyo, Japan) at 35°C until dry, and the residue was immediately dissolved in 10 mL of ethanol. Next, the solution was filtered through a 0.45-µm PTFE membrane syringe filter (Chromdisc, Daegu, Korea) for HPLC analysis. A 20 µL volume of the filtered sample was injected into a Waters Millenium system with a Waters 600 Controller gradient pump equipped with a degasser, a Waters 717 Plus autosampler, and a Waters UV-486 detector (Waters, MA). A SunFire C_18_ analytical column (2.6×250 mm, 5 µm, Waters, Ireland) was used, and the temperature was fixed at 30°C. The mobile phase was acetonitrile/methanol (75∶25, v/v) at a flow rate of 1.0 mL/min, and the detection wavelength was set to 264 nm. Vitamin D_2_ and ergosterol were determined by comparing the retention times with standards, and quantified according to a calibration curve.

### Experiment design

Ultraviolet irradiation optimization for vitamin D_2_ synthesis in oyster mushrooms was performed with RSM. The analysis used a factorial central composite rotator design (CCRD) for three factors with replicates at the center point. The variables included ambient temperature (A, °C), irradiation time (B, min), and irradiation intensity (C, W/m^2^), and the response variable was vitamin D_2_ content (µg/g, dry weight) in oyster mushrooms. In this study, the experiment design contained 18 trials, and the value of the responses was the mean of triplicate values. “Design Expert” (Version 8.0.6, Stat-Ease Inc., Minneapolis, MN, USA) statistical package was used to calculate the second-order polynomial coefficients, and analysis of variance (ANOVA) was evaluated to test the significance and adequacy of the model.

### Model verification

Optimal vitamin D_2_ synthesis conditions in oyster mushrooms based on ambient temperature, exposure time, and irradiation intensity were obtained according to the RSM predictive equations. The vitamin D_2_ content synthesized under predicted optimal conditions was investigated in triplicate, and the experimental and predicted values were compared to determine the validity of the model.

## Results and Discussion

### Selection of the design variables

**Figure 1:**
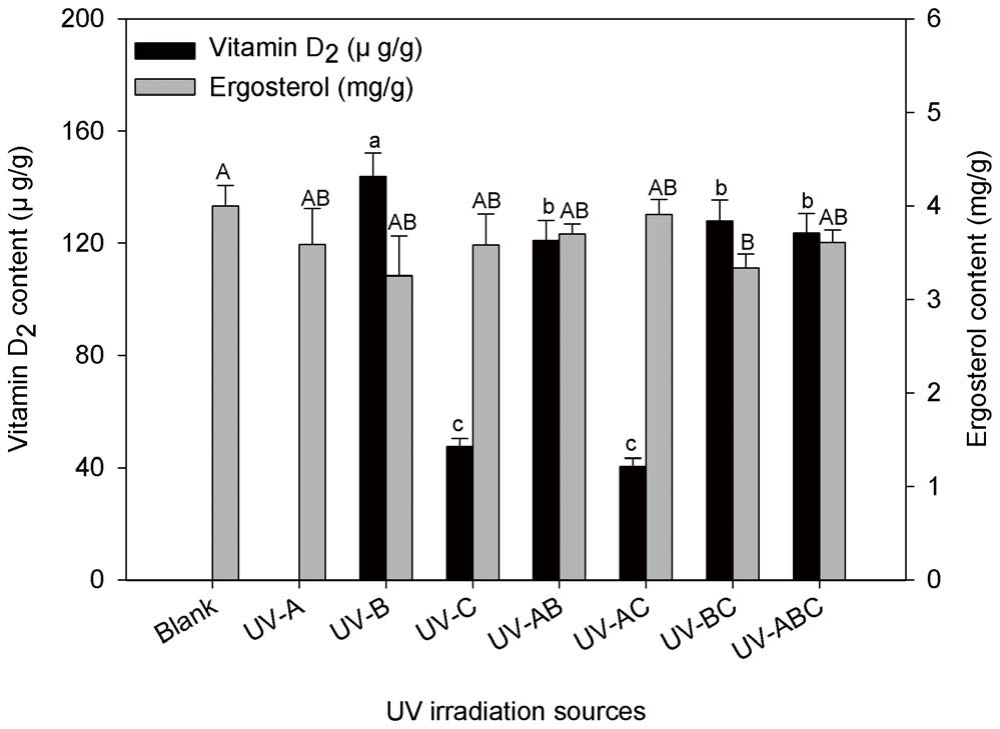
Effect of UV irradiation sources on the efficacy of vitamin D_2_ synthesis in oyster mushrooms. Results are expressed as the mean±SD of three replicates; Different small or capital letters are significantly different at *p*<0.01.

Previous studies used different UV irradiation sources to assess vitamin D_2_ formation in mushrooms. For example, Ko et al. [12] investigated the effect of UV-B in shiitake mushrooms, Koyyalamudi et al. [3] evaluated the effect of UV-C irradiation in button mushrooms, while Jasinghe and Perera [10] compared the effects of UV-A, UV-B, and UV-C. Additionally, two irradiation steps have been used to maximize vitamin D_2_ yield in the photochemical industry, first with the application of a lamp at 254 nm (UV-C), then with a lamp at 330–360 nm (UV-A) [17]. However, previous studies have not examined the effects of multiple UV sources on vitamin D_2_ efficiency in mushrooms. In this study, seven UV source combinations were used to determine the efficiency of vitamin D_2_ synthesis in oyster mushrooms, and the results are shown in Figure 1. The vitamin D_2_ concentration in blank (unexposed) and UV-A irradiation groups were too low to be detected (<2.0 µg/g), and these results were comparable to the findings of Teichmann et al [18]. However, the results of this study were different from a previous study by Jasinghe and Perera [10], which reported that mushrooms irradiated with UV-A (315–400 nm) experienced a significant increase in vitamin D_2_ content. This variance could be attributed to the different mushroom cultivators and different UV lamp manufacturers that were used in the studies. The low conversion rate of UV-A is possibly due to its lower level of penetration when compared to the more energetic UV-B and UV-C [6]. The vitamin D_2_ yields under sole UV-B irradiation (143.69 µg/g, dry weight) and the multiple irradiation sources, all of which contained UV-B exposures (UV-AB, UV-BC, and UV-ABC were 120.84, 127.89, and 123.58 µg/g, respectively) were significantly higher (*p*<0.01) than other irradiation sources. However, the ergosterol concentrations were slightly reduced by all types of UV irradiation, even though no significant difference was identified. These results indicated that vitamin D_2_ synthesis with a UV-B source of irradiation was much more efficient than with other irradiation sources, and these results were consistent with a previous study [10]. Moreover, it has been reported that effects of UV-B exposure on compositional changes of mushrooms were limited to significant increases in the vitamin D content, and no other nutritionally or toxicologically changes were identified [19]. Thus, UV-B irradiation was selected as the sole source to determine the optimal conditions for vitamin D_2_ synthesis in oyster mushrooms.

Besides of the wavelength of UV irradiation, the efficiency of vitamin D_2_ synthesis in mushrooms could be influenced by multiple parameters such as ambient temperature, exposure time, and irradiation intensity [3], [6], [10], [12], and the effects of these factors may be either independent or interactive. To optimize vitamin D_2_ synthesis conditions, a preliminary experiment was performed to determine the lower, middle and upper levels of each design variables that was used in the RSM. The results of the preliminary experiment are summarized in Table 1. According to the results, the vitamin D_2_ content enhanced with an increasing of temperature up to 35°C. These temperature effect profiles were similar to a previous study, which also reported that the highest yield of vitamin D_2_ occurred at 35°C, and that the decrease in the conversion rate beyond 35°C was likely due to heat stress (oxidative), browning pigment formation, further transformation of vitamin D_2_ as well as photo-degradation of irradiation [6]. Subsequently, the lower, middle and upper levels of the ambient temperature were selected as 25, 35, and 45°C, respectively. With regard to exposure time, the irradiation was performed at a temperature of 35°C, and the irradiation intensity was held at 0.6 W/m^2^. The results indicated that the vitamin D_2_ yield was increasing continuously with prolonged exposure times which ranged from 20 to 100 min, as shown in Table 1. The lower, middle and upper levels of exposure time that selected for RSM were 40, 80, and 120 min, respectively, in consideration of the practical applications of exposure time in industry. The vitamin D_2_ content dramatically increased when the irradiation intensity increased, whereas the analysis also demonstrated that the efficacy of ergosterol to vitamin D_2_ conversion was positively dependent on the irradiation intensity (Table 1). However, the adversely effects of irradiation are also important because high doses of irradiation could cause surface discoloration and moisture content decrease, which could affect the quality of mushrooms and influence the market value of the product [12]. Consideration of the cost-efficient, the results of the preliminary experiment allowed the investigators to select irradiation intensities at 0.6, 0.9, and 1.2 W/m^2^ for RSM optimization.

**Table 1:** Effects of independent variables on vitamin D_2_ synthesis in oyster mushrooms under UV-B irradiation.

Independent variables		Vitamin D_2_ (µg/g)	Ergosterol (mg/g)
Temperature (°C)	Blank	ND^c1)^	4.33±0.12^a^
	15	152.40±2.69^b^	4.24±0.22^ab^
	25	162.43±11.61^ab^	3.96±0.18^ab^
	35	178.38±5.96^a^	3.64±0.16^bc^
	45	151.12±15.50^b^	3.41±0.14^c^
Time (min)	20	137.63±11.45^c^	4.23±0.15^a^
	40	163.61±3.57^bc^	3.83±0.14^a^
	60	182.38±5.96^ab^	3.63±0.16^ab^
	80	194.26±28.70^a^	3.61±0.15^b^
	100	199.15±14.31^a^	3.52±0.14^b^
Intensity (W/m^2^)	0.3	144.61±2.37^b^	3.83±0.15^ab^
	0.6	180.53±0.91^a^	3.67±0.03^bc^
	0.9	197.58±12.6^a^	3.47±0.06^c^
	1.2	203.70±16.67^a^	3.20±0.08^c^

1) Values represent the mean±SD (*n* = 3). Means in each column with different letters are significantly different (*p*<0.01).

### Fitting the models

The ambient temperature, exposure time, and intensity of irradiation were identified as critical parameters for vitamin D_2_ synthesis in oyster mushrooms. A factorial central composite rotator design was employed to analyze the interactive effects of these parameters and determine the optimal level of each parameter. The experimental design and results are shown in Table 2. Multiple regression analysis of the experimental data provided a mathematical model that represented the vitamin D_2_ content in the experimental region. The relationship that was studied can be expressed by the following second-order polynomial model equation:
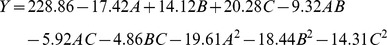
In this equation, *Y* is the predicted vitamin D_2_ content in oyster mushrooms (µg/g, dry weight); *A* is the ambient temperature; *B* is the exposure time; *C* is the irradiation intensity.

**Table 2:** Experimental design and central composite design results.

	Irradiation conditions				
Run	Temperature(°C)	Time (min)	Intensity (W/m^2^)	Vitamin D_2_ (µg/g)	Ergosterol (µg/g)
1	−1(25)	−1(40)	−1(0.6)	127.97±13.20	3.82±0.16
2	+1(45)	−1(40)	−1(0.6)	113.38±13.26	3.24±0.01
3	−1(25)	+1(120)	−1(0.6)	182.95±14.55	3.80±0.15
4	+1(45)	+1(120)	−1(0.6)	152.30±12.09	3.06±0.11
5	−1(25)	−1(40)	+1(1.2)	183.19±17.73	3.55±0.16
6	+1(45)	−1(40)	+1(1.2)	166.13±17.53	2.92±0.08
7	−1(25)	+1(120)	+1(1.2)	239.96±34.61	3.18±0.08
8	+1(45)	+1(120)	+1(1.2)	164.39±14.69	2.18±0.10
9	−α(18.2)	0(80)	0(0.9)	217.61±9.07	3.75±0.11
10	+α(51.8)	0(80)	0(0.9)	158.10±11.20	3.82±0.11
11	0(35)	−α(12.7)	0(0.9)	178.11±1.71	3.24±0.19
12	0(35)	+α(147.3)	0(0.9)	204.21±11.31	3.80±0.18
13	0(35)	0(80)	−α(0.4)	173.14±8.78	3.06±0.09
14	0(35)	0(80)	+α(1.4)	232.55±8.03	3.55±0.17
15	0(35)	0(80)	0(0.9)	240.54±13.91	2.92±0.16
16	0(35)	0(80)	0(0.9)	212.73±6.52	3.18±0.27
17	0(35)	0(80)	0(0.9)	226.64±8.31	3.08±0.13
18	0(35)	0(80)	0(0.9)	230.56±9.12	2.99±0.14

### Response surfaces analysis

Analysis of variance (ANOVA) was conducted to determine the significance of the fit of the second-order polynomial equation to the experimental data, which is shown in Table 3. The model *F*-value of 6.17 obtained in this study indicates that the model is significant. In addition, there is only a small chance (0.87%) that a “Model *F*-Value” of this size could occur due to noise. In this study, the *p*-value was less than 0.0100, which indicated that the model terms were significant. This study determined that A, B, C, A^2^, B^2^, and C^2^ were significant model terms, and that the irradiation intensity (C) was the considered as the most significant factor affecting vitamin D_2_ synthesis in oyster mushrooms. The model determination coefficient (*R* ^2^) was 0.8740, which indicated that the proposed model could account for 87% of the variability. The “lack-of-fit *F*-value” of 4.19 also indicates that lack of fit is not significant (*p* = 0.1341>0.05) relative to the pure error. Thus, the estimated models adequately fit the experimental data. In addition, the “Adeq. Precision” was used to measure the signal (response) to noise (deviation) ratio, and a ratio greater than 4 is indicative of an adequate signal. In this analysis the ratio of 6.979 indicated that there was an adequate signal, and that this model can be used to navigate the design space.

**Table 3:** ANOVA results for the response surface quadratic model.

Source	Sum of squares	DF1)	Mean square	*F*-value	*P*-value
Model	22043.57	9	2449.29	6.17	0.0087**
A-Temperature	4145.50	1	4145.50	10.44	0.0120*
B-Time	2722.13	1	2722.13	6.86	0.0307*
C-Intensity	5618.89	1	5618.89	14.15	0.0055**
AB	695.02	1	695.02	1.75	0.2224
AC	280.77	1	280.77	0.71	0.4248
BC	188.89	1	188.89	0.48	0.5099
A^2^	4862.31	1	4862.31	12.25	0.0081**
B^2^	4300.28	1	4300.28	10.83	0.0110*
C^2^	2589.12	1	2589.12	6.52	0.0340*
Residual	3176.63	8	397.08		
Lack of Fit	2778.38	5	555.68	4.19	0.1341
Pure Error	398.25	3	132.75		
Cor Total	25220.21	17			

1) Degrees of freedom.

*Significant at a level of 0.05; **Significant at a level of 0.01.

**Figure 2:**
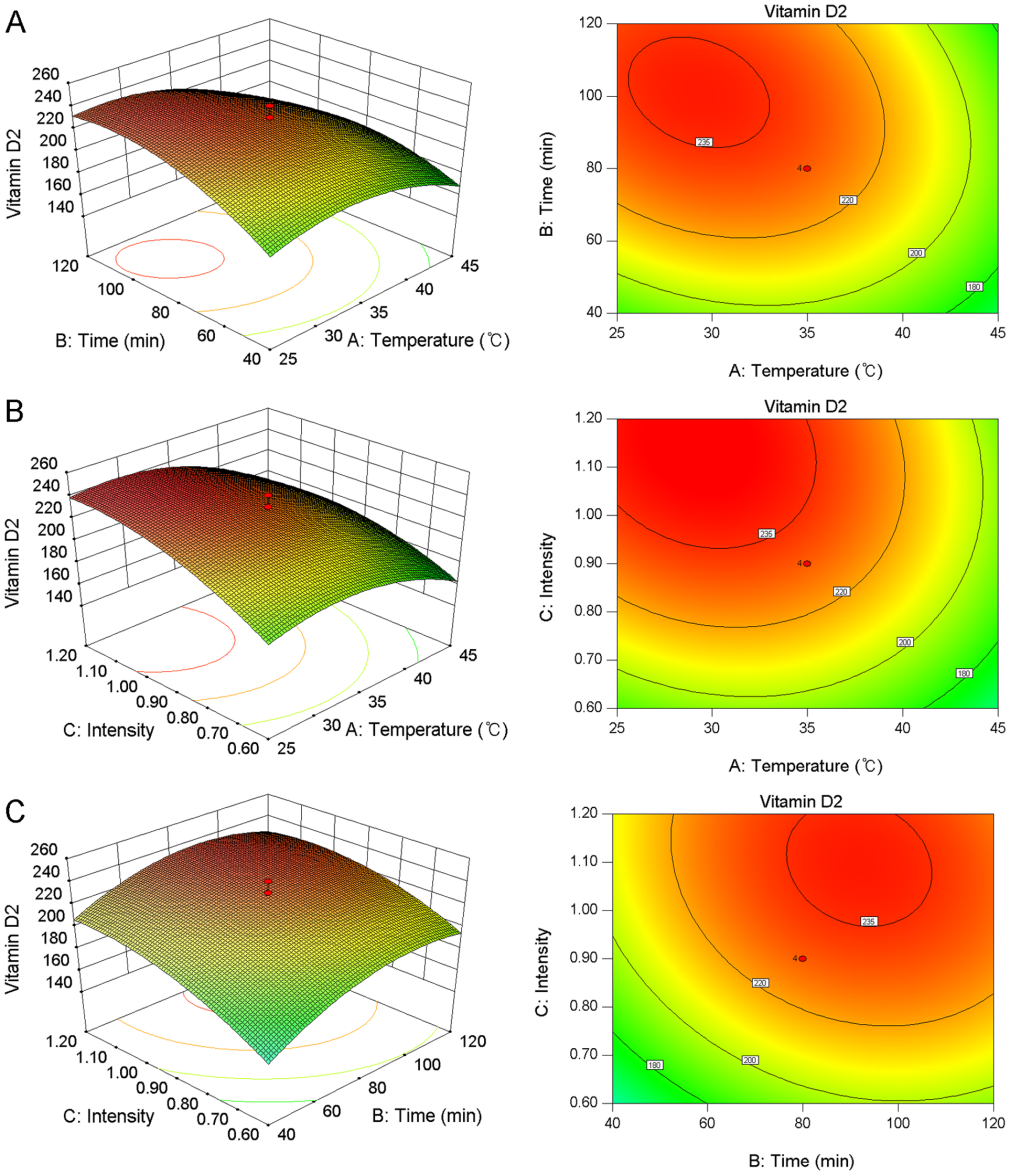
Response surface and contour plots for vitamin D_2_ production in oyster mushroom. **A**: Effects of ambient temperature and exposure time on vitamin D_2_ synthesis in oyster mushrooms at a constant UVB irradition intensity of 0.90 W/m^2^; **B**: Effects of ambient temperature and UVB irradition intensity on vitamin D_2_ synthesis in oyster mushrooms at a constant time period of 80 min; **C**: Effects of UVB irradition time and intensity on vitamin D_2_ synthesis in oyster mushrooms at a constant ambient temperature of 35°C.

To investigate the interaction among variables and to determine the optimal level of each factor for maximum vitamin D_2_ synthesis in oyster mushrooms, response surface plots and contour plots were obtained based on the model equation. Figure 2A depicts the effects of interactions that included ambient temperature (20–45°C) and exposure time (40–120 min) with a constant irradiation intensity of 0.9 W/m^2^. According to Figure 2A, both the ambient temperature and exposure time demonstrated quadratic effects on the response. The irradiation intensity caused a liner increase in the response under low levels of ambient temperature when the exposure time was maintained at 80 min, which were demonstrated in Figure 2B. In addition, the ambient temperature displayed a quadratic effect on the response and yielded maximum values between 28 and 32°C. Figure 2C depicts the interactions between exposure time and irradiation intensity, and shows a similar trend between the two variables. The analysis indicated that, with an increase in the length of exposure or the irradiation intensity, the vitamin D_2_ content synthesized by oyster mushrooms can be increased. Additionally, our results are consistent with previous reports identified that mushroom discoloration occurred with increased exposure time and irradiation intensity [3], which is considered to be undesirable for commercial production. This analysis determined that the optimal operation conditions for ambient temperature, exposure time, and UVB intensity were 28.16°C, 94.28 min, 1.14 W/m^2^, respectively. These optimal conditions produced the maximum amount of predicted vitamin D_2_ content of 245.49 µg/g, with a 95% confidence interval between 222.51 and 268.47 µg/g.

### Model verification

According to the results of the statistical design, the verification experiments were performed at the predicated conditions derived from ridge analysis of RSM, and that the vitamin D_2_ content synthesized in oyster mushrooms was at a remarkably high level of 239.67±4.53 µg/g (dry weight), which was within the 95% confidence interval of the maximum predicted value (245.49 µg/g). The correlation between the predicted and experimental values verified the validity and adequacy of the predicted model. The content of vitamin D_2_ achieved by the verified study was distinctly higher than the previous reported values (Table 4). In addition, no visible discoloration was identified by spectrophotometer (CM-3500d, Konica Minolta, Osaka, Japan) on the surface of the oyster mushrooms treated with these optimal conditions, which proposes that these parameters could be applied in commercial environments.

**Table 4:** Vitamin D_2_ content in irradiated mushrooms from different studies.

			Vitamin D_2_
Author and Year	Mushroom types	Treatment	(µg/g dry weight)
Mau et al. 1998 [11]	*Agaricus bisporus*	UV-C	7.30
Jasinghe and Perera 2005 [6]	*Pleurotus ostreatus*	UV-A	45.10
Jasinghe and Perera 2006 [10]	*Pleurotus ostreatus*	UV-B	184.00
Roberts et al. 2008 [13]	*Agaricus bisporus*	UV-B	7.98
Ko et al. 2008 [12]	*Lentinus edodes*	UV-B	61.90
Teichmann et al. 2007 [18]	*Cantharellus tubaeformis*	UV-C	14.03
Koyyalamudi et al. 2009 [3]	*Agaricus bisporus*	UV-C	23.13
Kalaras et al. 2011 [20]	*Agaricus bisporus*	Pulsed UV	27.00
Koyyalamudi et al. 2011 [21]	*Agaricus bisporus*	Pulsed UV	145.10
Simon et al. 2011 [19]	*Agaricus bisporus*	UV-B	54.10
Present study	*Pleurotus ostreatus* (fresh)	UV-B	239.67
	*Pleurotus ostreatus* (powder)	UV-B	498.10

### Production of vitamin D_2_ by lyophilized mushroom powder

**Figure 3:**
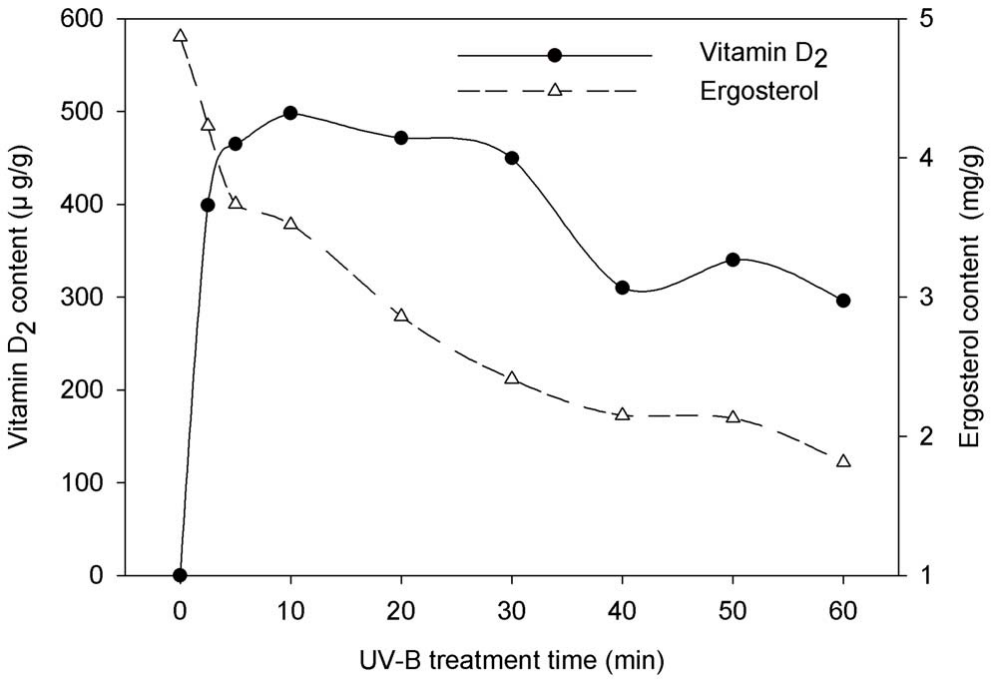
Conversion of ergosterol into vitamin D_2_ in homogenized mushroom powder. One gram of the lyophilized oyster mushroom powder (under 60-mesh) was spread over an area of 500 cm^2^ and subjected to UV-B irradiation for different time, at the intensity of 1.14 W/m^2^ and temperature of 28.16°C.

It is quite interesting to find out that not only fresh mushrooms but also lyophilized mushroom can produce vitamin D_2_ when exposed to UV-B lights. To increase the integral surface area, lyophilized mushroom was homogenized (under 60-mesh), then 1 g of the mushroom powder was spread over an area of 500 cm^2^ and subjected to UV-B irradiation at an intensity of 1.14 W/m^2^ and temperature of 28.16°C. The effect of UV-B exposure time on the conversion of ergosterol to vitamin D_2_ in lyophilized mushroom powder was represented in Figure 3. During the exposure, the ergosterol content was rapidly reduced; simultaneously vitamin D_2_ content was extremely increased and reached a maximum level of 498.10 µg/g within 10 min, then slightly decreased thereafter. It has been reported that irradiation contributes to an oxidative atmosphere causing photo-degradation of vitamin D_2_ due to prolong exposure [10]. In this study, the conversion of ergosterol to vitamin D_2_ was almost completed within 10 min, while prolonged exposure time decreased this conversion rate. When the conversion rate became unable to meet the photo-degradation rate (after 10 min), then resulted in a slight decrease of vitamin D_2_ content in mushroom powder as shown in Figure 3. Compared with fresh intact mushrooms, the homogenized mushroom powder remarkably increased the exposure surface area, result to an extremely increase of ergosterol conversion efficiency (Table 4).

## Conclusion

The present study confirmed that UV-B was the most suitable irradiation source for vitamin D_2_ synthesis in cultivated oyster mushrooms. Additionally, the efficiency of the vitamin D_2_ synthesis was influenced by ambient temperature, exposure time, and irradiation intensity. The factorial central composite rotator design was adopted to determine the optimal experimental conditions to enhance the vitamin D_2_ synthesis efficiency in oyster mushrooms. The proposed model equation illustrated the quantitative effect of variables in addition to the interactions of the variables on vitamin D_2_ synthesis. Based on the optimal conditions, the experimental vitamin D_2_ content of 239.67 µg/g (dry weight) was in very good agreements with the predicted value of 245.49 µg/g when irradiation performed at an ambient temperature of 28.16°C, UV-B intensity of 1.14 W/m^2^, and with an exposure time of 94.28 min. The results of this analysis indicate that these parameters can be used to optimize vitamin D_2_ synthesis in fresh oyster mushrooms for potential commercial application. Compared with irradiation on fresh mushrooms, UV-B treatment on lyophilized mushroom powder significantly improved the vitamin D_2_ content (498.10 µg/g) within much shorter exposure time (10 min), which could be used as a natural food additive for vitamin D_2_ fortified products.
